# Multicriteria decision making attributes and estimation of physicochemical properties of kidney cancer drugs via topological descriptors

**DOI:** 10.1371/journal.pone.0302276

**Published:** 2024-05-07

**Authors:** Mohamad Nazri Husin, Abdul Rauf Khan, Nadeem Ul Hassan Awan, Francis Joseph H. Campena, Fairouz Tchier, Shahid Hussain

**Affiliations:** 1 Special Interest Group on Modeling and Data Analytics, Faculty of Computer Science and Mathematics, Universiti Malaysia Terengganu, Nerus, Terengganu, Malaysia; 2 Department of Mathematics, Faculty of Sciences, Ghazi University, Dera Ghazi Khan, Pakistan; 3 Department of Mathematics and Statistics, De La Salle University, Malate Manila, Metro Manila, Philippines; 4 Mathematics Department, College of Science, King Saud University, Riyadh, Saudi Arabia; 5 Energy Engineering Division, Department of Engineering Science and Mathematics, Lulea University of Technology, Lulea, Sweden; Industrial University of Ho Chi Minh City, VIET NAM

## Abstract

Based on topological descriptors, QSPR analysis is an incredibly helpful statistical method for examining many physical and chemical properties of compounds without demanding costly and time-consuming laboratory tests. Firstly, we discuss and provide research on kidney cancer drugs using topological indices and done partition of the edges of kidney cancer drugs which are based on the degree. Secondly, we examine the attributes of nineteen drugs casodex, eligard, mitoxanrone, rubraca, and zoladex, etc and among others, using linear QSPR model. The study in the article not only demonstrates a good correlation between TIs and physical characteristics with the QSPR model being the most suitable for predicting complexity, enthalpy, molar refractivity, and other factors and a best-fit model is attained in this study. This theoretical approach might benefit chemists and professionals in the pharmaceutical industry to forecast the characteristics of kidney cancer therapies. This leads towards new opportunities to paved the way for drug discovery and the formation of efficient and suitable treatment options in therapeutic targeting. We also employed multicriteria decision making techniques like COPRAS and PROMETHEE-II for ranking of said disease treatment drugs and physicochemical characteristics.

## 1. Introduction

**Fig 1:**
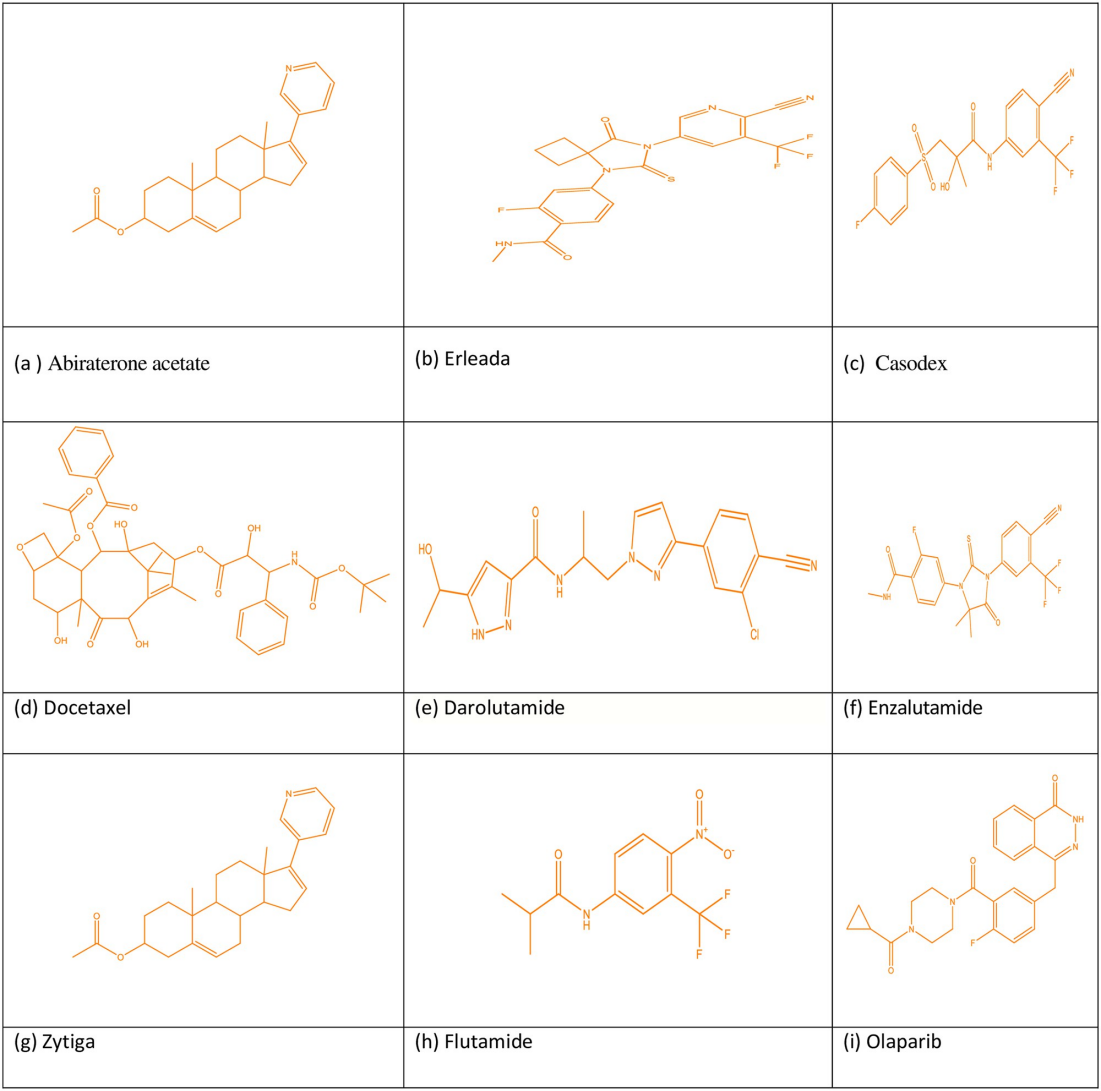
Kidney cancer drugs molecular structure.

**Fig 2:**
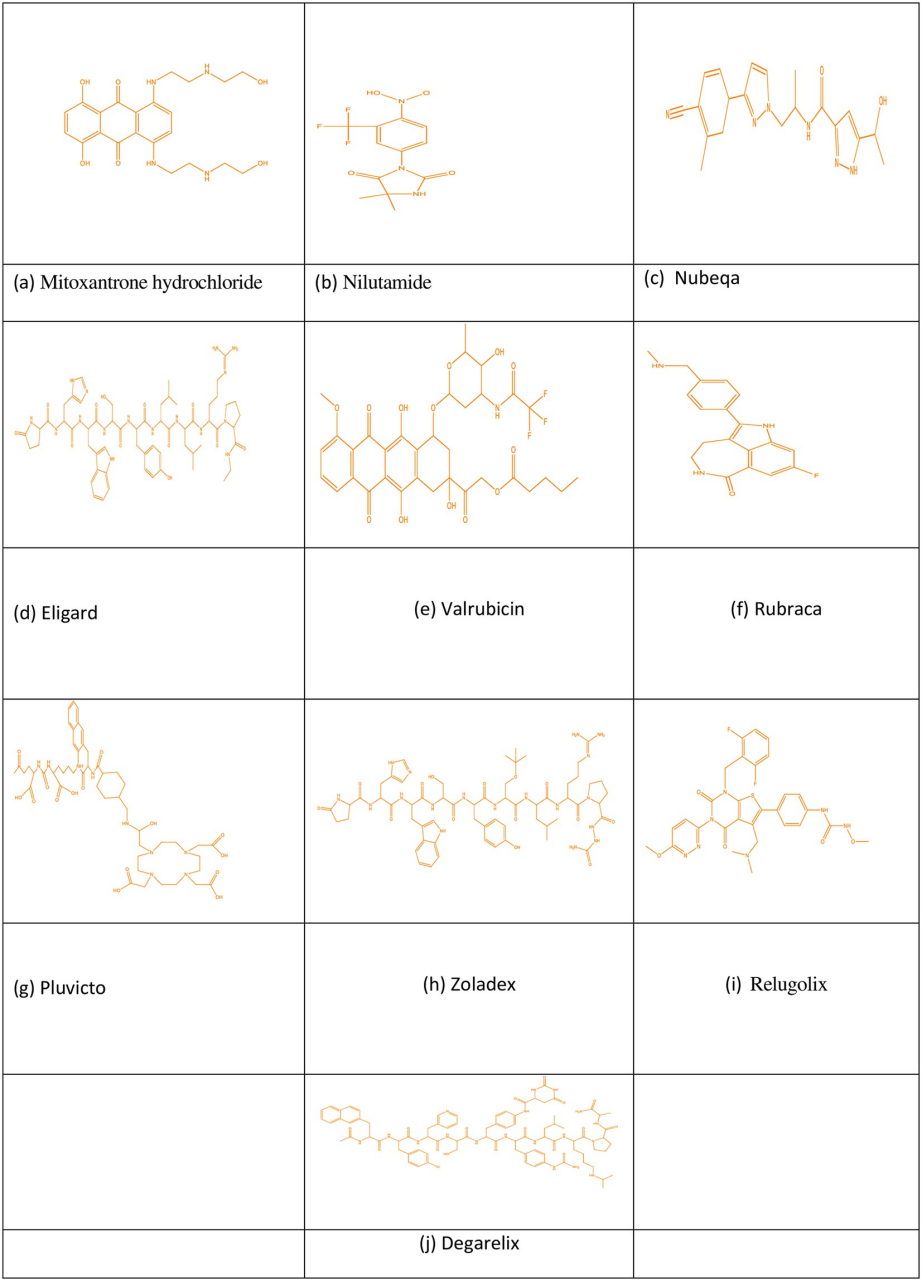
Molecular structure of kidney cancer drugs.

**Fig 3:**
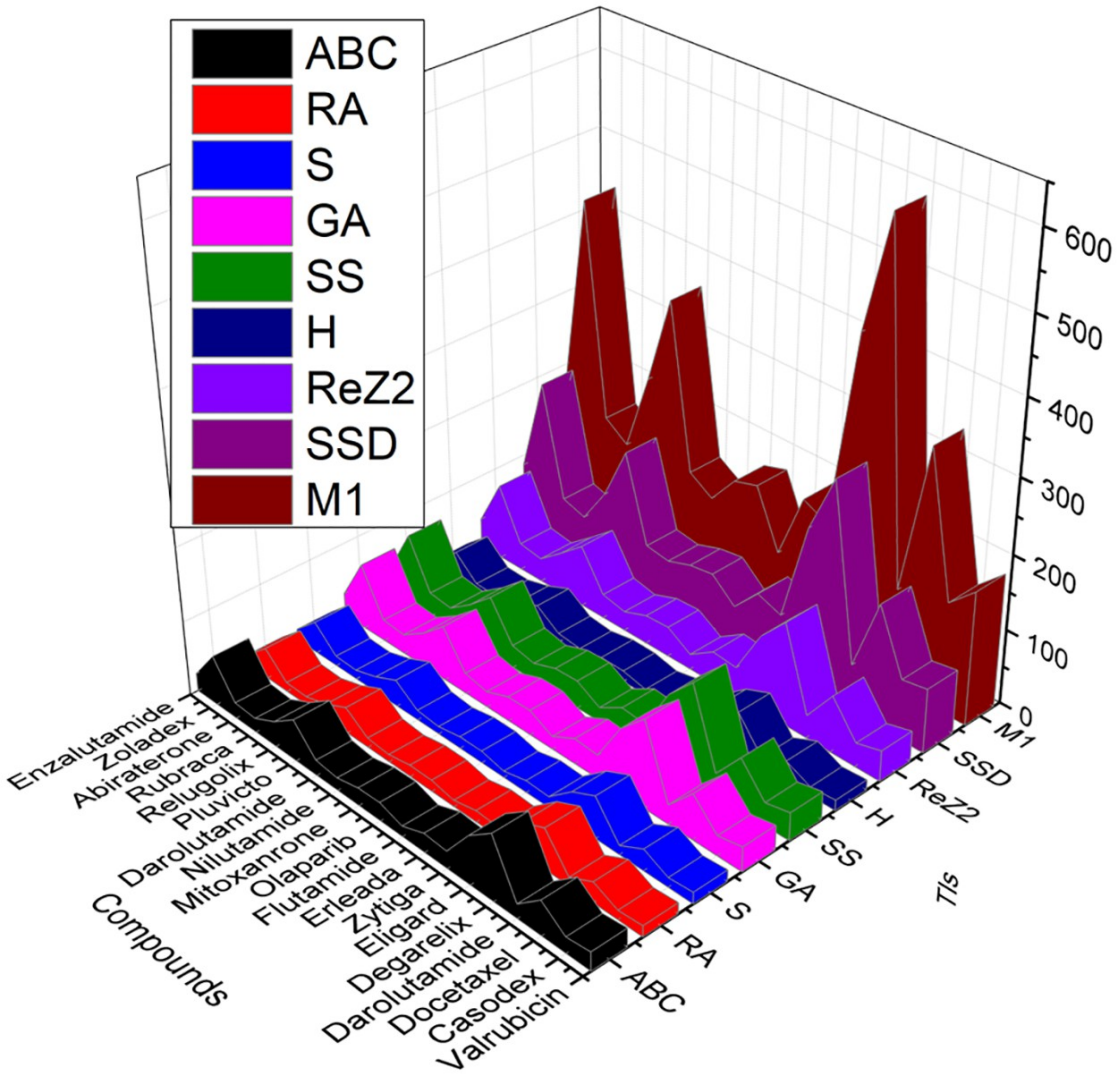
Graphical representation of kidney treatment drugs with TIs.

Healthy kidney cells grow out of control, resulting in renal cortical tumors and kidney cancer. It may be benign, slow-growing, or malignant. A malignant tumor is cancerous, which means it has the potential to develop and spread to other bodily regions. Although an indolent tumor can sometimes be malignant, it seldom metastasizes to other bodily regions. Kidney cancer develops in the renal tubule and collecting tubular epithelial system, which makes about 5% of all tumors. Major risk factors for kidney cancer include obesity, diabetes, hypertension, smoking, renal damage, and medication use. The main signs of kidney cancer were hematuria, renal discomfort, and mass. The disease’s early stages do not have any visible symptoms. As a result, people who want to receive medical treatment may already have kidney cancer that has spread to other parts of their bodies and may be experiencing related complications [1]. Malignant kidney tumors make up 2% of all cancer cases worldwide and are becoming more common. In the US, kidney cancer will see 63,000 new cases in 2018, 15,000 fatalities from the disease, and 350,000 new cases worldwide [2]. The condition known as kidney cancer is made up of a variety of distinct cancers, each of which has a unique histology, clinical course, response to treatment, and underlying genetic cause [3]. Over the previous 65 years, the rate of RCC has risen by 2% a year. This increase’s cause is not known. RCC makes up around 90% of renal cancers, while clear cell tumors make up 85% of these. Bellini (collecting) duct tumors, papillary tumors, and chromophore tumors are some additional, less frequent cell types. Less than 1% of all cases involve collecting duct cancer. The collecting duct renal cancer type medullary renal carcinoma was first mentioned in sickle cell trait-positive patients. Among the risk factors for developing RCC are smoking and obesity [4]. Transitional cell carcinoma is another name for this. It is the cause of 5% to 10% of adult kidney cancer diagnoses. This particular malignancy forms in the soft tissue of the kidney, the kidney’s thin capsule-like layer of connective tissue, or the surrounding fat. Surgery is typically used to treat renal sarcoma. The grade of a tumor generally refers to the extent of cell differentiation rather than the growth rate. Prompt and correct identification is crucial to improve the treatment of patients with kidney disease, reduce excess morbidity and death, limit the overuse of antimalarial drugs, and lessen the emergence of antimalarial drug resistance. Good diagnostic techniques must be employed in wealthy countries where there is usually a knowledge gap in kidney cancer and in resource-poor locations where malaria is a severe hazard to society. The mitigation or extinction of species is a problem for the scientific community. The illness claims a lot of lives on a global basis. New pharmaceuticals are developed and studied by scientists, and their discovery is problematic because it is expensive, time-consuming, and challenging in drug discovery. Numerous judgments are executed to extravagance, and stop said disease; for this, drug tests are carryout to combat the disease. It requires early verdicts and medication that will be suitable for the condition. The study of graphs with points and lines visually illustrating mathematical facts is a branch of mathematics and computer science. It can be used in a variety of situations. The application of graph theory has grown swiftly. Thoughtful the computer’s computation flow, communication networks, and data management are useful. The design of electrical connections, linguistics’ parsing of language trees, the syntax of a language tree, phonology, morphology, chemistry, physics, mathematics, and biology all depend heavily on graphs. The growth of theoretical chemistry relies heavily on graph theory. Topological indices (TIs) are the numerical representations of a molecular structure that the molecular graph provides. We may learn much about a molecule’s physicochemical and biological properties by using them in structure-property (QSPR) and structure-activity relationship (QSAR) investigations. In this research, potential antimalarial compounds, including valrubicin, casodex, docetaxel, darolutamide, degarelix, eligard, *zytiga*, erleada, flutamide, nubeqa, olaparib, mitoxanrone, nilutamide, pluvicto, relugolix, rubraca, abiraterone, zoladex and enzalutamide and other compounds are examined with TIs and linear regression modeling. Also, these medicines are harmless and operational, which is mandatory for the community. Figs 1–3 displays the drugs molecular structure. Some degree-based topological indices are built for these drugs’ chemical graphs.

Additionally, some of the physicochemical features of the drugs are estimated using TIs with the aid of QSPR facsimiles. Regression technique-acquired QSPR studies may aid in creating fresh medicines for the treatment of Kidney cancer. These indices and the antimalarial activity were discovered to be related. Other compound series do very well with this relationship. Therefore, the TIs could be vital in creating and synthesizing new drugs. Still, they must first be empirically validated, as this work’s conclusion demonstrates that both have a good relation. The ABC, Geometric, Zagreb, and Randic indices can be used to predict drug properties and bioactivity. In this article, we intended degree-based TIs on kidney cancer drugs. The said cancer drugs are carefully probed using TIs and imposed QSPR. It is found in this study that the regression model (RM) and TIs of drug properties have a good relationship. Through the analysis of topological indices, it is possible to discover molecular structure features in pharmaceutical sciences, which are crucial for creating novel products. In this study, the structure of a drug is represented as a graph so that vertex *V*(*G*) expresses an atom, and each edge *E*(*G*) represents a chemical connection between these atoms. All graphs are assumed to be simple and linked. The number of edges that connect a vertex to other edges determines its degree [5].

## 2. Material and methods

In this study, kidney cancer drugs are modeled by simple graphs. To compute the topological indices of the considered drug’s structure, the employed methods are vertex partitioning, edge partitioning, and computational techniques. In this study, we solely consider finite, simple, linked graphs. *G* is a graph with the vertex set *V* and the edge set *E*, assuming. The degree *d*_*u*_ of a vertex *u* depends on how many vertices are nearby. The list of topological formulas used in this study is provided below.

Zagreb index [6] is

$$M_{1}\left(G\right)=\sum_{uv\in E\left(G\right)}\left(d_{u}+d_{v}\right)$$ (1)

Randic index [7] is

$$RA\left(G\right)=\sum_{uv\in E\left(G\right)}\sqrt{\frac{1}{d_{u}d_{v}}}$$ (2)

The sum connectivity index [8] is

$$S\left(G\right)=\sum_{uv\in E\left(G\right)}\sqrt{\frac{1}{d_{u}+d_{v}}}$$ (3)

The geometric arithmetic index [9] is

$$GA\left(G\right)=\sum_{uv\in E\left(G\right)}\frac{2\sqrt{d_{u}d_{v}}}{d_{u}+d_{v}}$$ (4)

Redefined third Zagreb index [10] is

$$ReZG_{3}\left(G\right)=\sum_{uv\in E\left(G\right)}\left(d_{u}d_{v}\right)\left(d_{u}+d_{v}\right)$$ (5)

The harmonic index [20] of *G* is

$$H\left(G\right)=\sum_{uv\in E\left(G\right)}\frac{2}{d_{u}+d_{v}}$$ (6)

ABC index [11] *G* is

$$ABC\left(G\right)=\sum_{uv\in E\left(G\right)}\sqrt{\frac{d_{u+}d_{v}-2}{d_{u}d_{v}}}$$ (7)

SS index [12] *G* is

$$SS\left(G\right)=\sum_{uv\in E\left(G\right)}\sqrt{\frac{d_{u}d_{v}}{d_{u}+d_{v}}}$$ (8)

Symmetric division [13] is

$$SDD\left(G\right)=\sum_{uv\in E\left(G\right)}[\frac{d_{u}}{d_{v}}+\frac{d_{v}}{d_{u}}]$$ (9)

As an outcome, the RM model is preeminent to check and use for the examination above. Recently, Khan et al. [14] discussed bladder cancer drugs and applied a QSPR model to predict several disease drug properties. Previous research on covid-19 is deliberated by Colakoglu [15]. Havare discussed cancer treatment [16] and conversed that drug detection is an inflated and composite marvel that is best expected with this process. QSPR model of blood cancer drugs were done by Nasir et al. [17], which depicts the best-fit model. Developments in QSPR inspired us to work on existing research. This study aims to probe QSPR modeling of kidney disease drug regimens used in therapeutic management. The joint Rheumatoid arthritis (RA) investigation was done by Parveen et al. [18]. This encouraged us to study said cancer drug QSPR’s relation with TIs. Numerous experiments have discovered a clear connection between the molecular structures of chemical compounds and medications and their chemical properties, such as their boiling and melting points. Gao et al. [19] focused on a family of smart polymers frequently employed in creating anti-cancer medications. The results compensate for the lack of chemical and medical testing and serve as a theoretical framework for pharmaceutical engineering by determining the number of topological indices. The QSPR modeling of antituberculosis drugs is detailed in [20] completed the QSPR study of octane isomers and found a best-fit model for it. Earlier studies on covid-19, blood cancer, anti-cancer, and QSPR different TIs for various chemical structures motivate us much. Recently QSPR Analysis of varying skin cancer drugs are presented by Khan et al. in [21]. Sultana [22] probe into infertility drugs QSPR modeling in well versed way and made valuable contribution.

The mathematical description of Metal-Organic Networks (MONs) which are used in drug delivery, catalysis and sensing is provided [23]. In a number of scientific and technological domains fresh prospects are provided by a comprehensive closed mathematical description of graphyne and Zigzag graphyne nanoribbon [24]. Using the Laplacian polynomial, massive graphs with millions of vertices and edges are examined [25] and find modified results. The formulas for triangular cacti, boron nanotubes and their derivatives [26] as well as octagonal cell networks were obtained [27]. Numerous implications exist for Fuchsine acid in histology. QSPR evaluations were found [28] and prove elegant formulas. Reverse entropies in the critical volume and temperature analysis Henry’s law are substantial and can be calculated using the QSPR model [29]. For cerium oxide modified versions of the Hyper, Randić and Forgotten topological indices are examined [30]. The usefulness of Ve degree indices is covered [31] while SiO_4_ network’s temperature index is investigated [32] so as several effective predictors are defibrated [29] and insightful findings are made.

## 3 Mathematical computation of topological indices

This section represents TIs of kidney cancer drug and the QSPR modeling on drug molecular structures.

### 3.1 Computation of topological indices

Let *G* is a graph of mitoxantrone with edge partitions. |*E*_1,2_| = 2, |*E*_1,3_| = 4, |*E*_2,2_| = 12, |*E*_3,3_| = 10, |*E*_2,3_| = 6. By applying definitions 2.1 to 2.8, we get as follows:

$$\text{ABC}(G)=2\sqrt{\frac{1+2-2}{1\times 2}}+4\sqrt{\frac{1+3-2}{1\times 3}}+12\sqrt{\frac{2+2-2}{2\times 2}}+6\sqrt{\frac{2+3-2}{2\times 3}}\;+10\sqrt{\frac{3+3-2}{3\times 3}}\;=24.07$$

$$RA\left(G\;\right)=\;2\sqrt{\frac{1}{1\times 2}}+4\sqrt{\frac{1}{1\times 3}}\;+12\sqrt{\frac{1}{2\times 2}}\;+6\sqrt{\frac{1}{2\times 3}}\;\;+10\sqrt{\frac{1}{3\times 3}}\;=15.51$$

$$S\left(G\;\right)=2\sqrt{\frac{1}{1+2}}+\;\;4\sqrt{\frac{1}{1+3}}+12\sqrt{\frac{1}{2+2}}\;\;+6\sqrt{\frac{1}{2+3}}\;+10\sqrt{\frac{1}{3+3}}\;\;=15.92$$

$$\;GA\left(G\;\right)=\;\frac{4\sqrt{1\times 2}}{1+32}+\frac{8\sqrt{1\times 3}}{1+3}+\;\frac{24\sqrt{2\times 2}}{2+2}\;+\;\frac{12\sqrt{2\times 3}}{2+3}+\;\frac{20\sqrt{3\times 3}}{3+3}=33.23$$

$$H\left(G\;\right)=2\left(\frac{1}{1+2}\right)+4\left(\frac{1}{1+3}\right)+12\left(\frac{1}{2+2}\right)+6\left(\frac{1}{2+3}\right)+10\left(\;\frac{1}{3+3}\right)=15.07$$

$$SS\left(G\right)=2\sqrt{\frac{1\times 2}{1+2}}+4\sqrt{\frac{1\times 3}{1+3}}+12\sqrt{\frac{2\times 2}{2+2}}+6\sqrt{\frac{2\times 3}{2+3}}+10\sqrt{\frac{3\times 3}{3+3}}=35.92$$

$$SDD\left(G\right)=\left(\frac{1}{2}++\frac{2}{1}\right)+\left(\frac{1}{3}++\frac{3}{1}\right)+\left(\frac{2}{2}++\frac{2}{2}\right)+\left(\frac{2}{3}++\frac{3}{2}\right)+\left(\frac{3}{3}++\frac{3}{3}\right)=75.33$$

$$M_{1}\left(G\right)=2\left(1+2\right)+4\left(1+3\right)+12\left(2+2\right)+6\left(2+3\right)+10\left(3+3\right)=160$$

$$\;ReZG_{3}\left(G\;\right)=2\left(1\times 2\right)\left(1+2\right)+4\left(1\times 3\right)\left(1+3\right)+12\left(2\times 2\right)\left(2+2\right)+6\left(2\times 3\right)\left(2+3\right)+10\left(3\times 3\right)\left(3+3\right)=38.53$$

Topological indices of other drugs can be calculated using the identical technique with Eqs (1) to (9) given in Section 2. Table 1 mentions the TIs, and Figs 1–3 shows drugs and these may be found at Chemicalbook.

**Table 1:** Topological indices (Decision matrix).

Alternatives/Criteria	ABC	RA	S	GA	M_1_	SS	H	SDD	ReZG_3_
Valrubicin	0.1459	0.1422	0.144	0.1467	0.1578	0.1513	0.1405	0.1545	0.1556
Casodex	0.1255	0.1205	0.1191	0.1181	0.1298	0.1215	0.1157	0.1438	0.1208
Docetaxel	0.2718	0.266	0.2679	0.2716	0.2929	0.2799	0.2622	0.2906	0.2868
Darolutamide	0.1125	0.1131	0.1139	0.1146	0.114	0.1146	0.1134	0.1123	0.1147
Degarelix	0.5184	0.5285	0.5273	0.5253	0.5156	0.5214	0.5293	0.5193	0.5171
Eligard	0.3788	0.3897	0.3883	0.3866	0.3771	0.3829	0.3917	0.3767	0.3802
Zytiga	0.1357	0.1319	0.1362	0.1405	0.1473	0.144	0.1331	0.1364	0.1484
Erleada	0.1494	0.1473	0.1488	0.1513	0.1613	0.1556	0.1461	0.1569	0.1598
Flutamide	0.0825	0.0817	0.0793	0.0774	0.0824	0.0785	0.0783	0.0936	0.0772
Olaparib	0.1449	0.1455	0.1474	0.1492	0.1491	0.1498	0.1466	0.1432	0.1514
Mitoxanrone	0.139	0.147	0.1464	0.1462	0.1403	0.1441	0.1496	0.1343	0.1447
Nilutamide	0.0992	0.0943	0.0936	0.0937	0.1052	0.0975	0.09	0.1142	0.0986
Nubeqa	0.1213	0.1216	0.1222	0.1226	0.1228	0.1229	0.1214	0.1224	0.1228
Pluvicto	0.3235	0.3338	0.3303	0.3257	0.3139	0.3202	0.3342	0.3244	0.3136
Relugolix	0.1966	0.1962	0.2005	0.2053	0.2087	0.2082	0.1985	0.1931	0.2134
Rubraca	0.1102	0.1106	0.1134	0.1162	0.1158	0.1169	0.1128	0.1058	0.1193
Abiraterone	0.1229	0.1186	0.1234	0.1281	0.135	0.1317	0.1202	0.1227	0.1366
Zoladex	0.3835	0.3935	0.392	0.3903	0.3823	0.3871	0.3947	0.3833	0.3845
Enzalutamide	0.1396	0.1355	0.1355	0.1366	0.1491	0.1412	0.1319	0.1536	0.1441
Weights	0.1111	0.1112	0.1111	0.1111	0.1111	0.1111	0.1112	0.1111	0.1111
Maximum	89.74	55.77	57.36	119.41	588	129.95	53.33	291.33	137.67
Minimum	14.28	8.62	8.63	17.6	94	19.57	7.89	52.5	20.56

### 3.2 QSPR analysis

In this section, TIs are deliberated on kidney drugs. Calculation between QSPR and TIs shows that they are correlated. The potential drugs in Table 1 are used in a mathematical analysis that is used in kidney treatment. In this article, nine TIs for QSPR modeling drive are done. Properties such as molar refractivity (R), polar surface area (PSA), polarity (P), complexity (C), Surface Tension (ST), enthalpy (E), molar volume (MV), boiling point (BP), and flash point (FP) for nine cures used for kidney usage. The regression model (RM) imposed on drugs is tested by applying the equation. Using (10) the subsequent diverse linear models is helpful to find out other TIs and given below:

$$P=\alpha +\beta \;\left(X\right)$$ (10)

*P* is the property of the given drug. The *X* = *TI*, α is constant, and *β* is the regression coefficient. MATLAB and R-Language are used in the calculation. Nine TIs are used to analyze with the aid of linear QSPR. Eq (10) is relevant for results.

### 3.3 Linear regression models

In this section using (1), following linear regression models of TIs are mentioned Tables 2–10 shows parameters and QSPR models with TIs.

**Table 2:** Regression model of *ReZG*_3_(*G*).

Regression Models	N	R	r^2^	F
Molar Refractivity = -4.068+3.082 *ReZG*_3_(*G*)]	15	0.984	0.969	407.474
Polarity = 1.151+1.202 [*ReZG*_3_(*G*)]	15	0.967	0.934	170.756
Molar Volume = -10.012+8.071 [*ReZG*_3_(*G*)]	15	0.983	0.965	334.809
Complexity = -222.377+25.981 [*ReZG*_3_(*G*)]	15	0.982	0.965	472.201
Polar Surface Area = -33.345+3.770[*ReZG*_3_(*G*)]	15	0.922	0.85	73.946

**Table 3:** Regression model of RA (G).

Regression Models	N	R	r^2^	F
Molar refractivity = 4.691+7.627 [RA (G)]	15	.987	.973	475.323
Polarity = 4.461+2.966 [RA (G)]	15	.969	.938	183.030
Molar Volume = 11.789+21.609 [RA (G)]	15	.986	.972	412.933
Complexity = -130.491+62.369 [RA (G)]	15	.981	.962	426.579
Polar Surface Area = -24.772+9.453 [RA (G)]	15	.937	.877	92.788

**Table 4:** Correlation coefficients.

Topological Index	Correlation coefficients				
	Molar volume	Complexity	Polar Surface Area	Refractivity	Polarity
ABC(G)	.986	.983	.936	.986	.966
RA(G)	.986	.981	.937	.987	.969
S(G)	.986	.981	.932	.987	.970
GA(G)	.985	.981	.928	.987	.970
M_1_(G)	.983	.985	.927	.983	.962
SS(G)	.984	.983	.927	.986	.968
H(G)	.985	.979	.933	.987	.971
SDD(G)	.982	.986	.941	.978	.954
ReZG_2_(G)	.983	.982	.922	.684	.967

**Table 5:** Regression model of S (G).

Regression Models	N	R	r^2^	F
Molar refractivity = 3.296+7.421 [S (G)]	15	.987	.974	493.389
Polarity = 3.921+2.890 [S (G)]	15	.970	.941	189.886
Molar Volume = 8.990+20.992 [S (G)]	15	.986	.971	408.166
Complexity = -144.123+60.957 [S (G)]	15	.981	.962	430.208
Polar Surface Area = -25.692+9.152 [S (G)]	15	.932	.869	86.520

**Table 6:** Regression model of M_1_ (G).

Regression Models	N	R	r^2^	F
Molar Refractivity = -5.931+.723 [*M*_1_(*G*)]	15	.983	.966	367.433
Polarity = .541+.281 [*M*_1_(*G*)]	15	.962	.925	149.063
Molar Volume = -16.786+2.047 [*M*_1_(*G*)]	15	.983	.965	334.999
Complexity = -242.504+6.137 [*M*_1_(*G*)]	15	.985	.970	545.564
Polar Surface Area = -36.907+.891[*M*_1_(*G*)]	15	.927	.860	79.974

**Table 7:** Regression model of SS (G).

Regression Models	N	R	r^2^	F
Molar refractivity = -1.637+3.276 [SS (G)]	15	.986	.972	452.867
Polarity = 2.076+1.276 [SS (G)]	15	.968	.937	178.626
Molar Volume = -3.937+9.257 [SS (G)]	15	.984	.969	375.212
Complexity = -195.541+27.363 [SS (G)]	15	.983	.966	477.279
Polar Surface Area = -31.009+4.022 [SS (G)]	15	.927	.860	79.576

**Table 8:** Regression model of H (G).

Regression Models	N	R	r^2^	F
Molar refractivity = 5.869+7.960 [H (G)]	15	.987	.975	501.345
Polarity = 4.872+3.103 [H (G)]	15	.971	.943	197.925
Molar Volume = 16.208+22.515[H (G)]	15	.985	.971	403.651
Complexity = -118.010+64.820 [H (G)]	15	.979	.958	389.609
Polar Surface Area = -22.526+9.818 [H (G)]	15	.979	.958	389.609

**Table 9:** Regression model of SDD (G).

Regression Models	N	R	r^2^	F
Molar refractivity = -3.692+1.461 [SSD (G)]	15	.978	.957	291.997
Polarity = 1.443+.563 [SSD (G)]	15	.954	.910	121.805
Molar Volume = -14.862+4.163[SSD (G)]	15	.982	.964	319.194
Complexity = -222.065+12.357[SSD (G)]	15	.986	.972	589.309
Polar Surface Area = -37.483+1.835 [SSD (G)]	15	.941	.886	100.971

**Table 10:** Regression model of ABC (G).

Regression Models	N	R	r^2^	F
Molar refractivity = 0.310+4.760 [ABC (G)]	15	.986	.972	446.884
Polarity = 2.848+1.847[ABC (G)]	15	.966	.933	168.266
Molar Volume = -0.437+13.489 [ABC (G)]	15	.986	.971	408.881
Complexity = -174.928+39.508 [ABC (G)]	15	.983	.967	501.821
Polar Surface Area = -30.158+5.898 [ABC (G)]	15	.936	.875	91.148

### 3.3 Discussion

**Fig 4:**
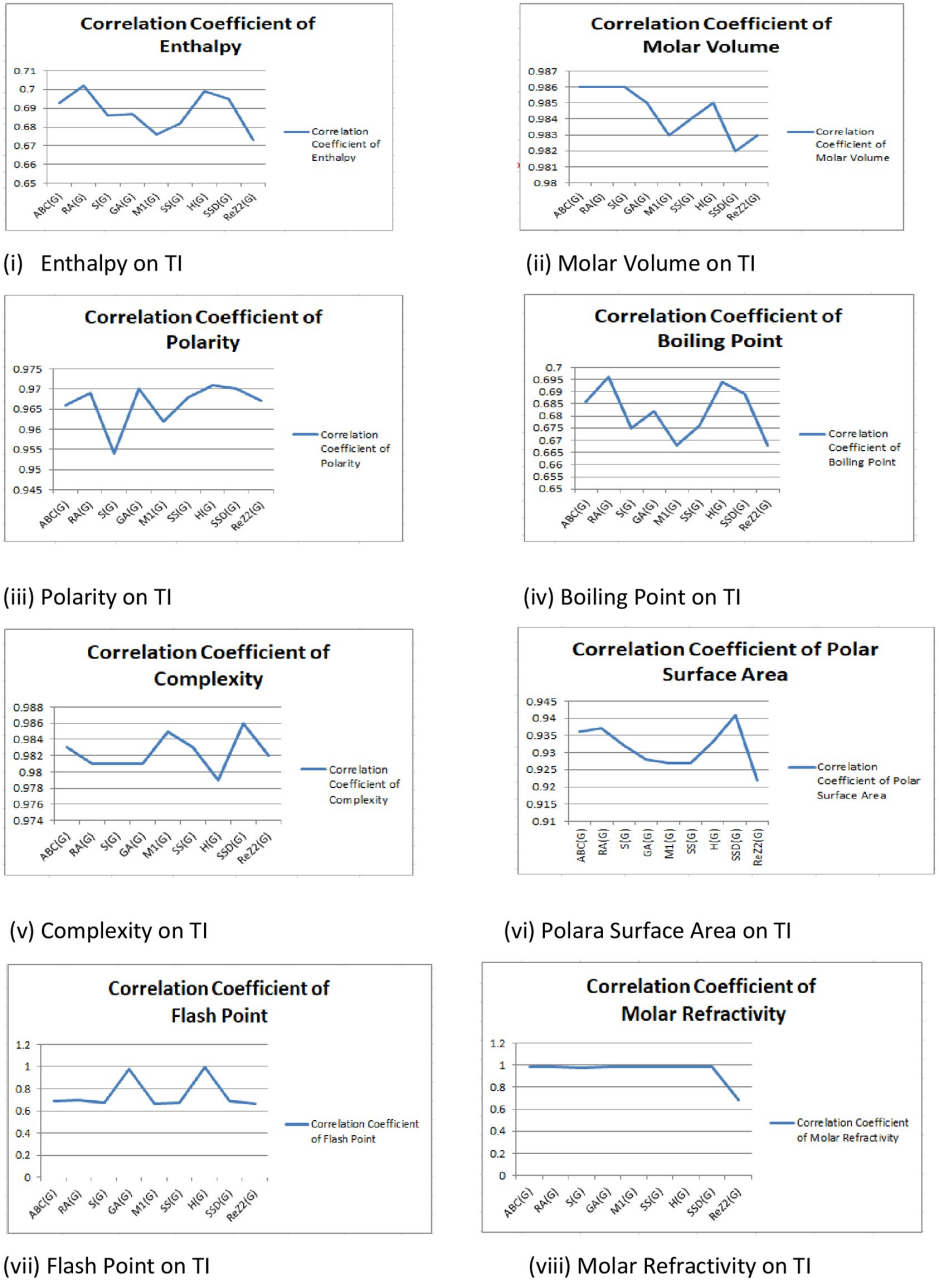
Correlation coefficient and TIs.

The main objective of this section is to establish a QSPR analysis between a number of TIs and to investigate a number of physicochemical properties and activities of medications, including valrubicin, casodex, docetaxel, darolutamide, degarelix, eligard, *zytiga*, erleada, flutamide, olaparib, mitoxantrone, nilutamide, pluvicto, nubeqa, relugolix, rubraca, abiraterone, zoladex and enzalutamide and probed effectiveness of these TIs. The numerical values of seven physicochemical characteristics are studied, including enthalpy (EP), flash point (FP), molecular volume (Mv), topological polar surface area (PSA), complexity (C), flash point (F), and boiling point (BP) etc. We obtained these values from PubChem. Table 4 lists the values of the correlation coefficients r between the physicochemical attributes and the defined degree-based topological indices. Also, the Tables 4–10 shows that there exist a best fit linear QSPR model and helpful to predict the properties. Parameters with model and TIs such as SDD (G) deliver maximum correlation of molar PSA r = 0.941 and complexity r = 0.986. GA (G), RA (G), S (G), and H (G) have r = 0.987 maximum refractivity. ABC (G), RA (G), and S (G) deliver maximum correlation of molar polarity r = 0.986. There exists a strong correlation except in surface tension and density. Table 4 shows no topological index exhibits a substantial correlation with surface tension and density. Fig 3 shows a graphic representation of the correlation and TIs. Medicines valrubicin, casodex, docetaxel, darolutamide, degarelix, eligard, *zytiga*, erleada, flutamide, olaparib, mitoxanrone, nilutamide, nubeqa, pluvicto, relugolix, rubraca, abiraterone, zoladex and enzalutamide etc. This trial can comrade and decide the augmentation of the model. Fig 4 depicts the graph between TIs and physical properties.

## 4. Multicriteria decision making attributes

This section uses a variety of topological indices to conduct a behavioral analysis of chemical structures. Chemical invariants are intended to give analysts and scientists an improved way of determining the chemical and physical properties of kidney cancer medications. Right now, we provide a weighted analysis that employs numerous topological indices. This weighted evaluation is carried out employing two alternative decision-making processes: Complex Proportional Assessment (COPRAS) and PROMETHEE (Preference Ranking Organization Method for Enrichment Evaluation) [33]. When many criteria are involved, COPRAS is a method presented by Zavadskas, Kaklauskas, and Sarka [34] for ranking and selecting options. While faced with a dearth of factors to consider, the PROMETHEE technique [35] offers helpful guidance as you make decisions. PROMETHEE II provides a thorough ranking, in contrast to PROMETHEE I, which only provides a partial ranking. The aforementioned improved technique is characterized by the control of incomparability throughout the entire ranking and the removal of scale impacts across criteria. Because it has a major impact on evaluation findings, the weight design is a crucial component of MCDM approaches. The weight of each criterion in this work is determined using the Entropy Method. To determine the weights of the criterion, the problem can be handled by applying the entropy technique. Information theory has incorporated it ever since its inception in the field of thermodynamics [36]. It can determine how much important information there is based on the data that is currently available. When items with the same indicator but a low entropy show a large variance, the indicator with the lower entropy provides more significant information, and the weight needs to be adjusted accordingly. The relative weight will decrease if the entropy increases and the discrepancy decreases. Thus, the entropy technique can be used to objectively measure weight [37].

### 4.1 COPRAS attribute

The well-known MCDM technique known as Complex Proportional Assessment (COPRAS) is applied in numerous disciplines [38]. The previously mentioned method determines the best and worst options from a range of possibilities. The main goal of the COPRAS technique is to assign multiple criteria to each alternative in order to rate them. The Tables 11–13 contains computed data for implementing COPRAS attribute and *Y*_*i*_ and *Qu*_*i*_ are computed by using following equations.


$$Z_{i}=H_{i+}+\frac{H_{i-\;min}\sum_{i}^{k}H_{i-}}{H_{i-\;min}\sum_{i=1}^{m}\frac{H_{i-min}}{H_{i-}}}$$

$$Y_{i}=\frac{Z_{i}}{Z_{i}\;max}\;\times 100$$

**Table 11:** Normalized evaluation matrix and entropy weights.

Alternatives/Criteria	ABC	RA	S	GA	M_1_	SS	H	SDD	REZG_3_
Valrubicin	0.1459	0.1422	0.144	0.1467	0.1578	0.1513	0.1405	0.1545	0.1556
Casodex	0.1255	0.1205	0.1191	0.1181	0.1298	0.1215	0.1157	0.1438	0.1208
Docetaxel	0.2718	0.266	0.2679	0.2716	0.2929	0.2799	0.2622	0.2906	0.2868
Darolutamide	0.1125	0.1131	0.1139	0.1146	0.114	0.1146	0.1134	0.1123	0.1147
Degarelix	0.5184	0.5285	0.5273	0.5253	0.5156	0.5214	0.5293	0.5193	0.5171
Eligard	0.3788	0.3897	0.3883	0.3866	0.3771	0.3829	0.3917	0.3767	0.3802
Zytiga	0.1357	0.1319	0.1362	0.1405	0.1473	0.144	0.1331	0.1364	0.1484
Erleada	0.1494	0.1473	0.1488	0.1513	0.1613	0.1556	0.1461	0.1569	0.1598
Flutamide	0.0825	0.0817	0.0793	0.0774	0.0824	0.0785	0.0783	0.0936	0.0772
Olaparib	0.1449	0.1455	0.1474	0.1492	0.1491	0.1498	0.1466	0.1432	0.1514
Mitoxanrone	0.139	0.147	0.1464	0.1462	0.1403	0.1441	0.1496	0.1343	0.1447
Nilutamide	0.0992	0.0943	0.0936	0.0937	0.1052	0.0975	0.09	0.1142	0.0986
Nubeqa	0.1213	0.1216	0.1222	0.1226	0.1228	0.1229	0.1214	0.1224	0.1228
Pluvicto	0.3235	0.3338	0.3303	0.3257	0.3139	0.3202	0.3342	0.3244	0.3136
Relugolix	0.1966	0.1962	0.2005	0.2053	0.2087	0.2082	0.1985	0.1931	0.2134
Rubraca	0.1102	0.1106	0.1134	0.1162	0.1158	0.1169	0.1128	0.1058	0.1193
Abiraterone	0.1229	0.1186	0.1234	0.1281	0.135	0.1317	0.1202	0.1227	0.1366
Zoladex	0.3835	0.3935	0.392	0.3903	0.3823	0.3871	0.3947	0.3833	0.3845
Enzalutamide	0.1396	0.1355	0.1355	0.1366	0.1491	0.1412	0.1319	0.1536	0.1441
Weights	0.1111	0.1112	0.1111	0.1111	0.1111	0.1111	0.1112	0.1111	0.1111

**Table 12:** Normalized weighted evaluation matrix.

Alternatives	Criteria								
Valrubicin	0.0139	0.0134	0.0132	0.0131	0.0144	0.0135	0.0129	0.016	0.0134
Casodex	0.0302	0.0296	0.0298	0.0302	0.0325	0.0311	0.0291	0.0323	0.0319
Docetaxel	0.0125	0.0126	0.0127	0.0127	0.0127	0.0127	0.0126	0.0125	0.0127
Darolutamide	0.0576	0.0587	0.0586	0.0584	0.0573	0.0579	0.0588	0.0577	0.0574
Degarelix	0.0421	0.0433	0.0432	0.043	0.0419	0.0425	0.0435	0.0419	0.0422
Eligard	0.0151	0.0147	0.0151	0.0156	0.0164	0.016	0.0148	0.0151	0.0165
Zytiga	0.0166	0.0164	0.0165	0.0168	0.0179	0.0173	0.0162	0.0174	0.0178
Erleada	0.0092	0.0091	0.0088	0.0086	0.0092	0.0087	0.0087	0.0104	0.0086
Flutamide	0.0161	0.0162	0.0164	0.0166	0.0166	0.0166	0.0163	0.0159	0.0168
Olaparib	0.0154	0.0163	0.0163	0.0162	0.0156	0.016	0.0166	0.0149	0.0161
Mitoxanrone	0.011	0.0105	0.0104	0.0104	0.0117	0.0108	0.01	0.0127	0.011
Nilutamide	0.0135	0.0135	0.0136	0.0136	0.0136	0.0136	0.0135	0.0136	0.0136
Nubeqa	0.0359	0.0371	0.0367	0.0362	0.0349	0.0356	0.0371	0.036	0.0348
Pluvicto	0.0218	0.0218	0.0223	0.0228	0.0232	0.0231	0.0221	0.0215	0.0237
Relugolix	0.0122	0.0123	0.0126	0.0129	0.0129	0.013	0.0125	0.0117	0.0133
Rubraca	0.0137	0.0132	0.0137	0.0142	0.015	0.0146	0.0134	0.0136	0.0152
Abiraterone	0.0426	0.0437	0.0436	0.0434	0.0425	0.043	0.0438	0.0426	0.0427
Zoladex	0.0155	0.0151	0.0151	0.0152	0.0166	0.0157	0.0147	0.0171	0.016

**Table 13:** Quantitative utility and ranking.

C_i+_	C_i-_	O_i_	Y_i_	Ranking
0.0573	0.0666	0.1904	72.8443	9
0.1278	0.1489	0.1873	71.6447	12
0.0506	0.0631	0.1911	73.1031	8
0.2303	0.2922	0.2606	99.7076	1
0.1685	0.2151	0.2097	80.2166	5
0.064	0.0753	0.1817	69.514	15
0.0704	0.0826	0.1777	67.9812	16
0.0368	0.0444	0.2366	90.5015	2
0.0659	0.0815	0.1746	66.804	17
0.0626	0.0809	0.1721	65.8348	18
0.0462	0.0523	0.2155	82.4618	3
0.0545	0.0677	0.1854	70.9418	13
0.1413	0.1831	0.1897	72.5714	10
0.0915	0.1108	0.1714	65.579	19
0.0508	0.0626	0.1924	73.6095	7
0.0584	0.0681	0.1885	72.1049	11
0.1707	0.2172	0.2115	80.9305	4
0.0653	0.0755	0.1827	69.8982	14
0.0493	0.0618	0.1928	73.7687	6

### 4.2 PROMETHEE-II

Brans invented the PROMETHEE technique, which includes PROMETHEE II for whole ranking of alternatives and PROMETHEE I for partial ranking [39]. In comparison to other multi-criteria analysis systems, this ranking method is extremely simple to design and use. The purpose of this is to assess and select a small number of options based on one or more criteria [40]. PROMETHEE requires two additional details. (i) Details regarding the level of relative importance, including the weights assigned to specific criteria. and (ii) Data pertaining to the comparative significance of each criterion, particularly the allocated weights. The information concerns decision-makers who are accustomed to independently evaluating the benefits of each choice in relation to every criterion. The weighted coefficients might be determined via the entropy method. We considered PROMETHEE II since we desired a detailed ranking of the possibilities.

For the beneficial criteria

$$H_{ij}=\frac{l_{ij}-\text{min}\;l_{ij}}{\text{max}\;l_{ij}-\text{min}\;l_{ij}}\;\text{for}\;j\;\in \left\{1,2,\ldots ,n\right\}\;and\;i\;\in \left\{1,2,\ldots ,m\right\}\;$$

For the non-beneficial criteria

$$H_{ij}=\frac{\text{max}\;l_{ij}-\;l_{ij}}{\text{max}\;l_{ij}-\text{min}\;l_{ij}}\;\text{for}\;j\;\in \left\{1,2,\ldots ,n\right\}\;and\;i\;\in \left\{1,2,\ldots ,m\right\}\;$$

Aggregated preference function

$$\varphi \left(\alpha ,\beta \right)=\frac{\sum_{j=1}^{n}W_{j}H_{ij}}{\sum_{j=1}^{n}W_{j}}$$

Tables 14 and 15 contain computational data related to PROMETHEE II.

**Table 14:** Normalized evaluation matrix.

Alternatives/Criteria	ABC	RA	S	GA	M_1_	SS	H	SDD	REZG_3_
**Valrubicin**	0.1455	0.1355	0.1443	0.1547	0.8259	0.8356	0.138	0.8569	0.8219
**Casodex**	0.0986	0.087	0.0889	0.0909	0.8907	0.9031	0.083	0.882	0.9009
**Docetaxel**	0.4344	0.4125	0.4209	0.4337	0.5142	0.5453	0.4078	0.5373	0.5234
**Darolutamide**	0.0689	0.0704	0.0772	0.083	0.9271	0.9185	0.0779	0.956	0.9147
**Degarelix**	1.0000	1.0000	1.0000	1.0000	0.0000	0.0000	1.000	0.000	0.0000
**Eligard**	0.6797	0.6895	0.6897	0.6904	0.3198	0.3128	0.695	0.335	0.3112
**Zytiga**	0.1222	0.1124	0.1268	0.1408	0.8502	0.8521	0.1215	0.8995	0.8381
**Erleada**	0.1535	0.147	0.1549	0.165	0.8178	0.8261	0.1503	0.8514	0.8123
**Flutamide**	0.0000	0.000	0.0000	0.000	1.0000	1.0000	0.0000	1.0000	1.0000
**Olaparib**	0.1431	0.1427	0.1519	0.1602	0.8462	0.8391	0.1514	0.8835	0.8314
**Mitoxanrone**	0.1297	0.1461	0.1496	0.1535	0.8664	0.8519	0.158	0.9044	0.8466
**Nilutamide**	0.0383	0.0282	0.0318	0.0362	0.9474	0.9571	0.026	0.9515	0.9513
**Nubeqa**	0.0891	0.0893	0.0956	0.1009	0.9069	0.8999	0.0955	0.9323	0.8963
**Pluvicto**	0.5529	0.5644	0.5602	0.5545	0.4656	0.4543	0.5673	0.4578	0.4626
**Relugolix**	0.2617	0.2564	0.2705	0.2856	0.7085	0.7073	0.2665	0.7662	0.6903
**Rubraca**	0.0635	0.0647	0.0761	0.0865	0.9231	0.9134	0.0766	0.9714	0.9043
**Abiraterone**	0.0926	0.0827	0.0983	0.1131	0.8785	0.8799	0.0929	0.9316	0.865
**Zoladex**	0.6906	0.698	0.6979	0.6986	0.3077	0.3032	0.7016	0.3196	0.3013
**Enzalutamide**	0.1311	0.1205	0.1254	0.1322	0.8462	0.8584	0.1188	0.859	0.848

**Table 15:** Ranking matrix.

Alternatives	Positive Flow μ^+^	Negative Flow μ^-^	Net out flow μ	Ranking
**Valrubicin**	0.0798	0.0042	0.0756	17
**Casodex**	0.0832	0.0044	0.0788	13
**Docetaxel**	0.1508	0.0079	0.1429	5
**Darolutamide**	0.0929	0.0049	0.088	8
**Degarelix**	0.4112	0.0216	0.3895	1
**Eligard**	0.2554	0.0134	0.2419	3
**Zytiga**	0.0801	0.0042	0.0759	16
**Erleada**	0.0819	0.0043	0.0775	15
**Flutamide**	0.1186	0.0062	0.1123	6
**Olaparib**	0.0847	0.0045	0.0802	12
**Mitoxanrone**	0.0882	0.0046	0.0835	10
**Nilutamide**	0.0744	0.0039	0.0705	19
**Nubeqa**	0.0881	0.0046	0.0835	11
**Pluvicto**	0.2048	0.0108	0.1941	4
**Relugolix**	0.1099	0.0058	0.1041	7
**Rubraca**	0.0925	0.0049	0.0876	9
**Abiraterone**	0.0823	0.0043	0.0779	14
**Zoladex**	0.259	0.0136	0.2454	2
**Enzalutamide**	0.0788	0.0041	0.0746	18

## 5. Conclusion

In this study, we estimated TIs for medications used to treat kidney cancer and compared them to the QSPR model. Theoretical inferences from this article’s findings are advantageous for developing new kidney cancer drugs. Our findings reveal a distinct trend in investigating structures and their physical characteristics.

1. ABC, RA and S index gave best results of molar volume with r value 0.986.
2. Complexity can be best estimated with SSD index with r value 0.986.
3. Polar surface area is best results with Randic index.
4. Refractivity is best assessed with r value 0.987 of RA, S, GA and H and polarity with H index having r value 0.971

**Fig 5:**
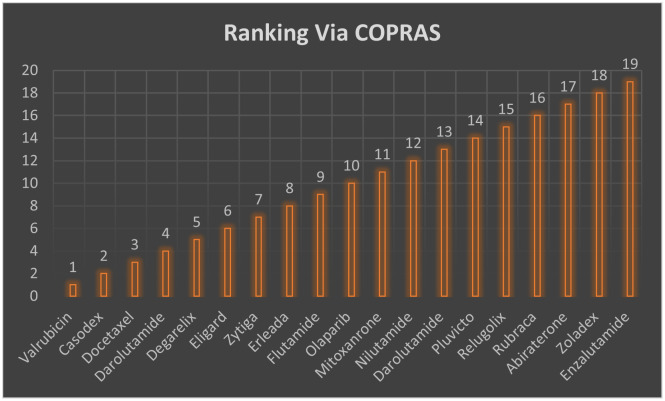
Ranking of kidney treatment drugs via COPRAS.

**Fig 6:**
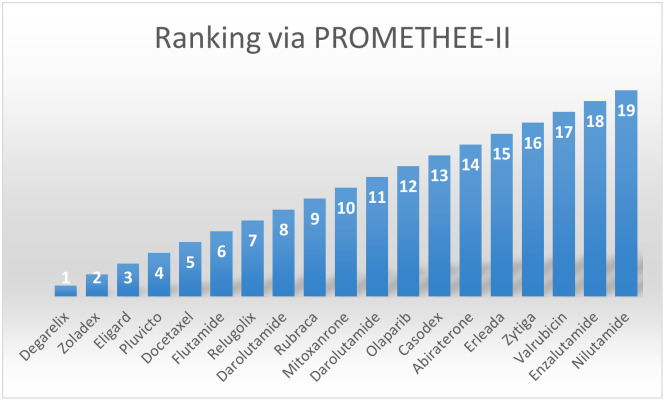
Ranking of kidney treatment drugs via PROMETHEE-II.

Finally, the study helps to design new kidney drugs efficiently and preventive measures for the disease mentioned above. QSPR and TI’s premeditated tenets are eye-opening to chemists deployed on drug-determining phenomena in the pharmaceutical industry. They provide new approaches in estimating properties for specific diseases and drugs, but it is essentially validated, as this work’s conclusion demonstrates. In this study, we also employed two multicriteria decision making techniques like COPRAS and PROMETHEE-II to make ranking of the medicines used for kidney cancer treatment as shown in Figs 5 and 6, respectively.
