# Correction: Multicriteria decision making attributes and estimation of physicochemical properties of kidney cancer drugs via topological descriptors

**DOI:** 10.1371/journal.pone.0305486

**Published:** 2024-06-12

**Authors:** Mohamad Nazri Husin, Abdul Rauf Khan, Nadeem Ul Hassan Awan, Francis Joseph H. Campena, Fairouz Tchier, Shahid Hussain

There are errors in the Funding statement. The correct Funding statement is as follows: This work was supported by the Universiti Malaysia Terengganu under the Interdisciplinary Impact Driven Research Grant (ID^2RG) 2023, vote no. 55516. Researchers Supporting Project number (RSP2024R401), King Saud University, Riyadh, Saudi Arabia.
